# Tripotential Differentiation of Adherently Expandable Neural Stem (NS) Cells

**DOI:** 10.1371/journal.pone.0000298

**Published:** 2007-03-14

**Authors:** Tamara Glaser, Steven M. Pollard, Austin Smith, Oliver Brüstle

**Affiliations:** 1 Institute of Reconstructive Neurobiology, LIFE & BRAIN Center, University of Bonn and Hertie Foundation, Bonn, Germany; 2 Wellcome Trust Centre for Stem Cell Research, University of Cambridge, Cambridge, United Kingdom; Netherlands Cancer Institute, Netherlands

## Abstract

**Background:**

A recent study has shown that pure neural stem cells can be derived from embryonic stem (ES) cells and primary brain tissue. In the presence of fibroblast growth factor 2 (FGF2) and epidermal growth factor (EGF), this population can be continuously expanded in adherent conditions. In analogy to continuously self-renewing ES cells, these cells were termed ‘NS’ cells (Conti et al., PLoS Biol 3: e283, 2005). While NS cells have been shown to readily generate neurons and astrocytes, their differentiation into oligodendrocytes has remained enigmatic, raising concerns as to whether they truly represent tripotential neural stem cells.

**Methodology/Principal Findings:**

Here we provide evidence that NS cells are indeed tripotent. Upon proliferation with FGF2, platelet-derived growth factor (PDGF) and forskolin, followed by differentiation in the presence of thyroid hormone (T3) and ascorbic acid NS cells efficiently generate oligodendrocytes (∼20%) alongside astrocytes (∼40%) and neurons (∼10%). Mature oligodendroglial differentiation was confirmed by transplantation data showing that NS cell-derived oligodendrocytes ensheath host axons in the brain of myelin-deficient rats.

**Conclusions/Significance:**

In addition to delineating NS cells as a potential donor source for myelin repair, our data strongly support the view that these adherently expandable cells represent *bona fide* tripotential neural stem cells.

## Introduction

Neural stem cells are defined as clonogenic cells capable of self-renewal and multipotent differentiation into the three principle cell types of the CNS – neurons, astrocytes and oligodendrocytes. They have been isolated from the fetal [1]–[6] and adult [7]–[14] mammalian central nervous system (CNS). Another source of neural stem cells are embryonic stem (ES) cells [15], [16]. In the adult brain, the subventricular zone (SVZ) of the lateral ventricles, which generates olfactory bulb neurons, and the subgranular zone (SGZ) of the hippocampus are the primary regions where neurogenesis occurs [8], [11], [17], [18]. Fetal and adult neural stem cells have been shown to exhibit properties of radial glia and astrocytes, respectively [19]–[24]. Neural stem cells have been frequently propagated as neurospheres, multicellular aggregates which proliferate in the presence of epidermal growth factor (EGF) and/or fibroblast growth factor 2 (FGF2) [7], [25]. Upon plating and differentiation, they give rise to neurons, astrocytes and oligodendrocytes. However, neurospheres are limited in that they contain a mixture of neural stem cells and more differentiated progenitor cells in a common extracellular matrix [26]–[28]. Clonal analyses of dissociated single sphere cells revealed that only a small percentage (3–4%) of the cells within neurospheres are truly multipotent stem cells [29], [30].

Survival, proliferation and differentiation of stem cells appear to be regulated by both cell-autonomous and environmental signals [31], [32]. Intrinsic regulators include proteins involved in asymmetric cell division, nuclear factors controlling gene expression and epigenetic modifications. In vivo, the external signals that control stem cell fate collectively make up the stem cell ‘niche’ [33], [34]. This niche has powerful effects on their resident stem cells in maintaining a balance of quiescence, self-renewal, and cell fate commitment. Signals generated from the niche include a wide range of secreted factors, cell-cell interactions mediated by integral membrane proteins and the extracellular matrix. Neurosphere cultures are supposed to provide some of these niche signals that may be relevant for neural stem cell maintenance, survival and proliferation.

In a recent study Conti et al. have reported on niche independent symmetrical self renewal of adherently growing neural stem cells derived from primary CNS tissue and ES cells [35]. These cells are diploid and clonogenic and undergo sustained symmetrical self-renewal divisions in response to FGF2 and EGF independent from any specific cellular niche. In analogy to self-renewing pluripotent ES cells, they were termed ‘NS’ cells. NS cells were found to express Pax6, GLAST and BLBP mRNAs and are immunopositive for nestin, RC2, vimentin, 3CB2, SSEA1/Lex1, Pax6 and prominin. These markers are considered to be diagnostic for neurogenic radial glia, suggesting that NS cells are closely related to a radial glia lineage [36]. NS cells also express the neural precursor markers Sox2, Sox3, and Emx2, and the bHLH transcription factors Olig2 and Mash1.

Upon exposure to serum or BMP4, NS cells differentiate into astrocytes. Culture without EGF followed by FGF2 withdrawal gives rise to cells with immunochemical and electrophysiological properties of mature neurons. Importantly, even after prolonged expansion, NS cells maintain their potential to differentiate efficiently into neurons and astrocytes in vitro and upon transplantation into the adult brain. However, the culture conditions used so far did not support the differentiation of NS cells into oligodendrocytes.

In the past, growth and differentiation factors suitable for the proliferation and differentiation of oligodendocyte progenitors have been successfully used to derive myelinating oligodendrocytes from ES cells [37], [38]. We set out to explore whether these paradigms support tripotential differentiation of NS cells including the oligodendroglial lineage.

## Results and Discussion

**Figure 1:**
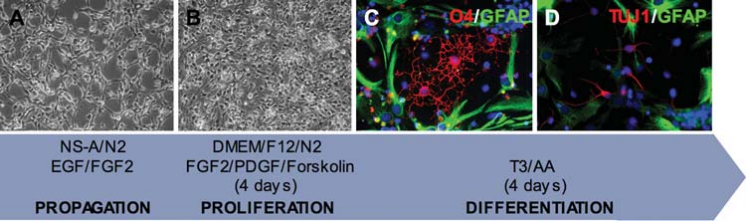
Protocol for the generation of oligodendrocytes from NS cells. NS cells propagated in NS-A medium plus N2 in the presence of EGF and FGF2 (A) were cultured in DMEM/F12 plus N2 in the presence of FGF2, PDGF and forskolin for 4 days on polyornithine/laminin coated plastic (B) before they were induced to differentiate by growth factor withdrawal in the presence of 3,3,5-tri-iodothyronine hormone (T3) and ascorbic acid (AA) (C,D). After four days, immunostaining for the O4 antigen revealed differentiation into oligodendrocytes (C). The differentiated cultures also contained GFAP-positive astrocytes and ß-III tubulin/TUJ1-positive neurons (C,D), demonstrating the tripotential differentiation capacity of these cells.

**Figure 2:**
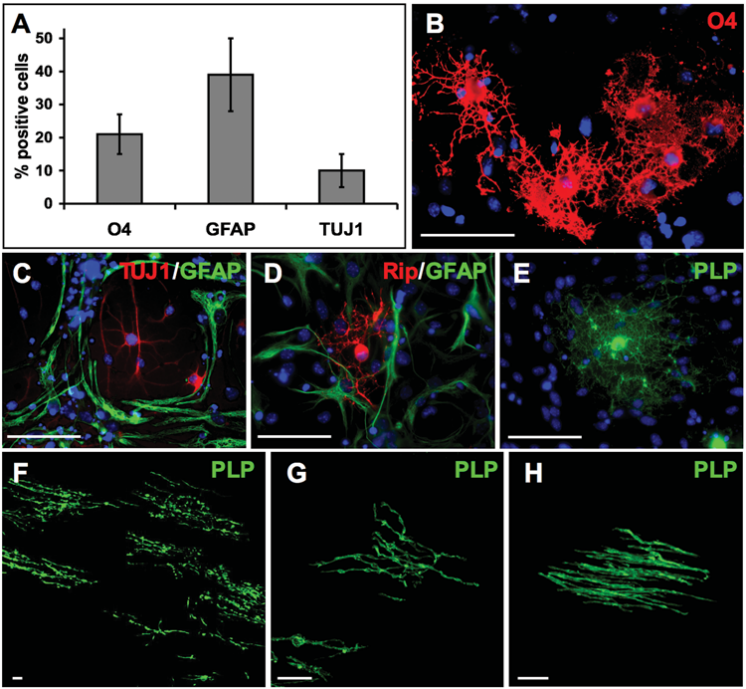
Tripotential differentiation of NS cells in vitro and generation of myelinating oligodendrocytes in vivo. (A–E) Quantitative marker expression and representative immunofluorescence images. The specific culture conditions used to differentiate NS cells resulted in the generation of oligodendrocytes (∼20%) positive for O4 (B), Rip (D) and PLP (E), GFAP-expressing astrocytes (∼40%; C–D) and neurons positive for ß-III tubulin/TUJ1 (∼10%; C). (F–H) NS cells cultured in N2 medium and proliferated for 4 days in the presence of FGF2, PDGF and forskolin were transplanted into the brain of 2- to 3-day-old myelin-deficient rats. Two weeks after transplantation, the engrafted cells had formed PLP-positive myelin internodes. Shown are representative pictures from septum (F) and corpus callosum (G–H). Scale bars B–D, 100 µm; F–H, 20 µm.

The clonally derived NS cell line NS-5 generated from mouse ES cells was propagated and passaged according to Conti et al. [35] (**Fig. 1A**). To promote oligodendroglial differentiation cells were cultured on polyornithine/laminin coated dishes in medium containing N2 supplement plus FGF2, platelet-derived growth factor (PDGF) and forskolin, a growth factor combination known to enhance oligodendrocyte progenitor proliferation. After 4 days this condition resulted in a population of small cells with condensed cell bodies and short processes (**Fig. 1B**). Terminal differentiation was initiated by a 4-day-growth factor withdrawal in the presence of the thyroid hormone tri-iodothyronine (T3) and ascorbic acid [38]. Under these conditions, NS cells efficiently differentiated into oligodendrocytes, astrocytes and neurons (**Fig. 1C,D**). Quantitative results of the antigenic marker expression are summarized in **Fig. 2A**. Differentiated cultures contained 21±4% O4-positive oligodendrocytes (**Fig. 2B**), 45±9% of the cells expressed the astrocytic marker GFAP (**Fig. 2C,D**) and a fraction of 11±3% was found to be positive for the neuronal antigen ß-III tubulin/TUJ1 (**Fig. 2C**). Oligodendroglial differentiation could also be demonstrated by labelling with the RIP antibody, which recognizes 2′,3′-cyclic nucleotide 3′-phosphodiesterase (CNPase) in oligodendrocytes [39] (**Fig. 2D**). Furthermore, cells with ramified processes and characteristic oligodendroglial morphology expressed myelin proteolipid protein (PLP) (**Fig. 2E**). Similar data have been obtained with NS cells derived from foetal mouse brain (Sandra G. Lopez & Steven M. Pollard, data not shown).

To study whether NS cells can generate oligodendrocytes in vivo, we transplanted them into the brain of 2- to 3-day-old myelin-deficient (md) rats. This mutant carries a point mutation in the PLP gene, which results in severe dysmyelination and oligodendrocyte cell death [40]. Due to the lack of endogenous myelin formation and the absence of PLP expression in md rats, donor-derived internodes can be easily detected by mere PLP immunolabeling [37], [38]. Following injection into the cerebral hemispheres, donor cells propagated in the presence of FGF2, PDGF and forskolin migrated into the host tissue where they adopted highly ramified morphologies with parallel PLP-positive processes typical of mylinating oligodendrocytes (**Fig. 2F–H**). Two weeks after transplantation, donor-derived PLP-positive myelin internodes were found in a variety of host brain regions including septum (**Fig. 2F**), striatum and corpus callosum (**Fig. 2G,H**).

Taken together, these data demonstrate that the differentiation spectrum of NS cells is not restricted to neurons and astrocytes but extends also to oligodendrocytes. This multipotential differentiation capacity of ES-cell-derived NS cells confirms their status of a tripotent stem cell. Previous studies have shown that tissue- and ES cell-derived oligodendrocyte progenitors can be efficiently proliferated in the presence of FGF2 and PDGF [37], [41]. Furthermore, T3 and ascorbic acid are known to promote the differentiation and survival of oligodendrocytes [6], [38]. Thus, the mechanisms regulating primary oligodendrocyte progenitor proliferation and differentiation appear to be preserved in NS cells. Importantly, the ability for oligodendroglial differentiation is maintained after transplantation. Our data show that NS cells proliferated in FGF2 and PDGF can migrate into the recipient brain, integrate with host cells and ensheath host axons in a myelin-deficient environment. These observations not only support the ability of NS cells to generate mature oligodendrocytes but also depict them as a potential donor source for myelinating transplants.

## Materials and Methods

### Culture and differentiation of NS cells

ES cell-derived NS cells were generated and cultured as previously described [35]. All experiments were done on the clonal cell line NS-5. For oligodendroglial differentiation cells were plated on polyornithin/laminin-coated dishes, and the NS cell expansion medium, which is composed of NS-A medium (Euroclone, Pero, Italy) plus N2 supplement (Invitrogen; Karlsruhe, Germany), was replaced by DMEM/F12 supplemented with N2 (N2 medium). Cells were proliferated in the presence of FGF2 (10 ng/µl; R&D Systems, Wiesbaden, Germany), PDGF (10 ng/µl; R&D Systems) and forskolin (10 µM; Sigma, Steinheim, Germany) for 4 days to support a glial precursor stage. Differentiation was induced by a 4-day-growth factor withdrawal in the presence of 3,3,5-tri-iodothyronine (T3; 30 ng/µl; Sigma) and ascorbic acid (AA; 200 µM; Sigma).

### Immunocytochemical analysis

Cell cultures were fixed with 4% PFA for 10 min at room temperature. After washing in PBS, cells were blocked with 5% normal goat serum in PBS for 15 min and incubated over night in 1% normal goat serum in PBS with the following primary antibodies: O4 (mouse IgM; 1∶10; Chemicon), Rip (mouse IgG; 1∶100; Developmental Studies Hybridoma Bank, University of Iowa, Iowa City, IA), proteolipid protein (PLP) (rabbit IgG; 1∶500; provided by I.R. Griffiths, Institute of Comparative Medicine, Glasgow, Scottland), GFAP (rabbit IgG; 1∶100; DAKO, Hamburg, Germany) and ß-III tubulin (mouse IgG; clone TUJ1; 1∶500; Babco/Covance, Richmond, CA). For intracellular antigens cells were permeabilized in PBS containing 0.1% Triton X-100. Antigens were visualized using appropriate fluorochrome-conjugated secondary antibodies applied at 1∶250 for 1h (goat anti-mouse IgG-Cy3, goat anti-mouse IgM-Cy3, goat anti-rabbit IgG-FITC; Jackson Immuno Research; West Baltimore Pike, PA, USA). DAPI was used for nuclear counterstaining. Labeled cells were preserved in Vectashield (Vector Laboratories) and analysed using a Zeiss fluorescence microscope. Negative controls for antibody specificity were performed by omitting the primary antibodies. Quantitative analysis was carried out by counting the number of immunoreactive cells per total number of viable cells as determined by DAPI staining. Data for each marker are based on triplicate cultures with ≥20 randomly chosen high power fields quantified for each staining.

### Cell transplantation, tissue processing and analysis

Cell transplantation into the brain of early postnatal (P2–P3) myelin-deficient rats (md rats; kindly provided by Ian Duncan, University of Wisconsin, Madison, USA) was performed as described [42]. Briefly, cells proliferating in FGF2 and PDGF for 4 days were harvested with trypsin-EDTA (0.5 mg/ml; Sigma), followed by treatment with soybean trypsin inhibitor (0.5 mg/ml; Sigma). They were concentrated in Hanks' buffered salt solution and injected into the frontal part of the right and left hemisphere (200.000 cells/injection in a total volume of 2 µl). Cells were injected through the skin and meninges using a flame-polished glass micropipette of 75 µm internal diameter. Fourteen days following transplantation, the recipients were anesthetized with a mixture of xylazine (10 mg/kg) and ketamine (80 mg/kg) and perfused with 4% paraformaldehyde in PBS. The transplanted brains were post-fixed at 4°C over night, rinsed in PBS, cryoprotected in 30% sucrose for at least 3 days and cut in an freezing microtome in the coronal plane. To identify engrafted oligodendrocytes, 40 µm cryostat sections were subjected to an anti-PLP immunofluorescence analysis. All recipient animals (n = 9) were housed under standard laboratory conditions, and the surgical procedures were performed in accordance with institutional guidelines.

## References

[1] Davis AA, Temple S (1994). A self-renewing multipotential stem cell in embryonic rat cerebral cortex.. Nature.

[2] Kilpatrick TJ, Bartlett PF (1993). Cloning and growth of multipotential neural precursors: requirements for proliferation and differentiation.. Neuron.

[3] Reynolds BA, Tetzlaff W, Weiss S (1992). A multipotent EGF-responsive striatal embryonic progenitor cell produces neurons and astrocytes.. J Neurosci.

[4] Uchida N, Buck DW, He D, Reitsma MJ, Masek M (2000). Direct isolation of human central nervous system stem cells.. Proc Natl Acad Sci U S A.

[5] Vescovi AL, Parati EA, Gritti A, Poulin P, Ferrario M (1999). Isolation and cloning of multipotential stem cells from the embryonic human CNS and establishment of transplantable human neural stem cell lines by epigenetic stimulation.. Exp Neurol.

[6] Johe KK, Hazel TG, Muller T, Dugich-Djordjevic MM, McKay RD (1996). Single factors direct the differentiation of stem cells from the fetal and adult central nervous system.. Genes Dev.

[7] Reynolds BA, Weiss S (1992). Generation of neurons and astrocytes from isolated cells of the adult mammalian central nervous system.. Science.

[8] Lois C, Alvarez-Buylla A (1993). Proliferating subventricular zone cells in the adult mammalian forebrain can differentiate into neurons and glia.. Proc Natl Acad Sci U S A.

[9] Gritti A, Parati EA, Cova L, Frolichsthal P, Galli R (1996). Multipotential stem cells from the adult mouse brain proliferate and self-renew in response to basic fibroblast growth factor.. J Neurosci.

[10] Weiss S, Dunne C, Hewson J, Wohl C, Wheatley M (1996). Multipotent CNS stem cells are present in the adult mammalian spinal cord and ventricular neuroaxis.. J Neurosci.

[11] Palmer TD, Takahashi J, Gage FH (1997). The adult rat hippocampus contains primordial neural stem cells.. Mol Cell Neurosci.

[12] Palmer TD, Markakis EA, Willhoite AR, Safar F, Gage FH (1999). Fibroblast growth factor-2 activates a latent neurogenic program in neural stem cells from diverse regions of the adult CNS.. J Neurosci.

[13] Johe KK, Hazel TG, Muller T, Dugich-Djordjevic MM, McKay RD (1996). Single factors direct the differentiation of stem cells from the fetal and adult central nervous system.. Genes Dev.

[14] Johansson CB, Momma S, Clarke DL, Risling M, Lendahl U (1999). Identification of a neural stem cell in the adult mammalian central nervous system.. Cell.

[15] Tropepe V, Hitoshi S, Sirard C, Mak TW, Rossant J (2001). Direct neural fate specification from embryonic stem cells: a primitive mammalian neural stem cell stage acquired through a default mechanism.. Neuron.

[16] Ying QL, Stavridis M, Griffiths D, Li M, Smith A (2003). Conversion of embryonic stem cells into neuroectodermal precursors in adherent monoculture.. Nat Biotechnol.

[17] Doetsch F, Caille I, Lim DA, Garcia-Verdugo JM, Alvarez-Buylla A (1999). Subventricular zone astrocytes are neural stem cells in the adult mammalian brain.. Cell.

[18] Doetsch F (2003). A niche for adult neural stem cells.. Curr Opin Genet Dev.

[19] Gotz M (2003). Glial cells generate neurons–master control within CNS regions: developmental perspectives on neural stem cells.. Neuroscientist.

[20] Gotz M, Steindler D (2003). To be glial or not-how glial are the precursors of neurons in development and adulthood?. Glia.

[21] Merkle FT, Tramontin AD, Garcia-Verdugo JM, Alvarez-Buylla A (2004). Radial glia give rise to adult neural stem cells in the subventricular zone.. Proc Natl Acad Sci U S A.

[22] Doetsch F (2003). The glial identity of neural stem cells.. Nat Neurosci.

[23] Goldman S (2003). Glia as neural progenitor cells.. Trends Neurosci.

[24] Mori T, Buffo A, Gotz M (2005). The novel roles of glial cells revisited: the contribution of radial glia and astrocytes to neurogenesis.. Curr Top Dev Biol.

[25] Reynolds BA, Weiss S (1996). Clonal and population analyses demonstrate that an EGF-responsive mammalian embryonic CNS precursor is a stem cell.. Dev Biol.

[26] Singec I, Knoth R, Meyer RP, Maciaczyk J, Volk B (2006). Defining the actual sensitivity and specificity of the neurosphere assay in stem cell biology.. Nat Methods.

[27] Campos LS, Leone DP, Relvas JB, Brakebusch C, Fassler R (2004). Beta1 integrins activate a MAPK signalling pathway in neural stem cells that contributes to their maintenance.. Development.

[28] Reynolds BA, Rietze RL (2005). Neural stem cells and neurospheres–re-evaluating the relationship.. Nat Methods.

[29] Gritti A, Frolichsthal-Schoeller P, Galli R, Parati EA, Cova L (1999). Epidermal and fibroblast growth factors behave as mitogenic regulators for a single multipotent stem cell-like population from the subventricular region of the adult mouse forebrain.. J Neurosci.

[30] Tropepe V, Sibilia M, Ciruna BG, Rossant J, Wagner EF (1999). Distinct neural stem cells proliferate in response to EGF and FGF in the developing mouse telencephalon.. Dev Biol.

[31] Watt FM, Hogan BL (2000). Out of Eden: stem cells and their niches.. Science.

[32] Morrison SJ, Shah NM, Anderson DJ (1997). Regulatory mechanisms in stem cell biology.. Cell.

[33] Li L, Xie T (2005). Stem cell niche: structure and function.. Annu Rev Cell Dev Biol.

[34] Fuchs E, Tumbar T, Guasch G (2004). Socializing with the neighbors: stem cells and their niche.. Cell.

[35] Conti L, Pollard SM, Gorba T, Reitano E, Toselli M (2005). Niche-independent symmetrical self-renewal of a mammalian tissue stem cell.. PLoS Biol.

[36] Pollard SM, Conti L, Sun Y, Goffredo D, Smith A (2006). Adherent neural stem (NS) cells from fetal and adult forebrain.. Cereb Cortex.

[37] Brustle O, Jones KN, Learish RD, Karram K, Choudhary K (1999). Embryonic stem cell-derived glial precursors: a source of myelinating transplants.. Science.

[38] Glaser T, Perez-Bouza A, Klein K, Brustle O (2005). Generation of purified oligodendrocyte progenitors from embryonic stem cells.. Faseb J.

[39] Watanabe M, Sakurai Y, Ichinose T, Aikawa Y, Kotani M (2006). Monoclonal antibody Rip specifically recognizes 2′,3′-cyclic nucleotide 3′-phosphodiesterase in oligodendrocytes.. J Neurosci Res.

[40] Boison D, Stoffel W (1989). Myelin-deficient rat: a point mutation in exon III (A—C, Thr75—Pro) of the myelin proteolipid protein causes dysmyelination and oligodendrocyte death.. Embo J.

[41] Bogler O, Wren D, Barnett SC, Land H, Noble M (1990). Cooperation between two growth factors promotes extended self-renewal and inhibits differentiation of oligodendrocyte-type-2 astrocyte (O-2A) progenitor cells.. Proc Natl Acad Sci U S A.

[42] Klein D, Schmandt T, Muth-Kohne E, Perez-Bouza A, Segschneider M (2006). Embryonic stem cell-based reduction of central nervous system sulfatide storage in an animal model of metachromatic leukodystrophy.. Gene Ther.

